# Considering the Experimental Use of Temozolomide in Glioblastoma Research

**DOI:** 10.3390/biomedicines8060151

**Published:** 2020-06-04

**Authors:** Verena J. Herbener, Timo Burster, Alicia Goreth, Maximilian Pruss, Hélène von Bandemer, Tim Baisch, Rahel Fitzel, Markus D. Siegelin, Georg Karpel-Massler, Klaus-Michael Debatin, Mike-Andrew Westhoff, Hannah Strobel

**Affiliations:** 1 Department of Pediatrics and Adolescent Medicine, University Medical Center Ulm, D-89075 Ulm, Germany; verena.herbener@uni-ulm.de (V.J.H.); alicia.goreth@uni-ulm.de (A.G.); helenevonbandemer@gmail.com (H.v.B.); tim.baisch@uni-ulm.de (T.B.); rahel.fitzel@uni-ulm.de (R.F.); klaus-michael.debatin@uniklinik-ulm.de (K.-M.D.); hannah.strobel@uniklinik-ulm.de (H.S.); 2 Department of Biology, School of Sciences and Humanities, Nazarbayev University, Nur-Sultan 010000, Kazakhstan; timo.burster@nu.edu.kz; 3 Department of Gynecology and Obstetrics, Medical Faculty, University Hospital of the Heinrich-Heine-University Duesseldorf, D-40225 Duesseldorf, Germany; maximilian.pruss@med.uni-duesseldorf.de; 4 Department of Neurosurgery, University Medical Center Ulm, D-89081 Ulm, Germany; georg.karpel@uniklinik-ulm.de; 5 Department of Pathology and Cell Biology, Columbia University Medical Center, New York, NY 10032, USA; ms4169@cumc.columbia.edu

**Keywords:** Glioblastoma, limitations of experimental systems, established cell lines, Temozolomide

## Abstract

Temozolomide (TMZ) currently remains the only chemotherapeutic component in the approved treatment scheme for Glioblastoma (GB), the most common primary brain tumour with a dismal patient’s survival prognosis of only ~15 months. While frequently described as an alkylating agent that causes DNA damage and thus—ultimately—cell death, a recent debate has been initiated to re-evaluate the therapeutic role of TMZ in GB. Here, we discuss the experimental use of TMZ and highlight how it differs from its clinical role. Four areas could be identified in which the experimental data is particularly limited in its translational potential: 1. transferring clinical dosing and scheduling to an experimental system and vice versa; 2. the different use of (non-inert) solvent in clinic and laboratory; 3. the limitations of established GB cell lines which only poorly mimic GB tumours; and 4. the limitations of animal models lacking an immune response. Discussing these limitations in a broader biomedical context, we offer suggestions as to how to improve transferability of data. Finally, we highlight an underexplored function of TMZ in modulating the immune system, as an example of where the aforementioned limitations impede the progression of our knowledge.

## 1. Introduction

When investigating potential drugs for their clinical merit, the necessary experiments cannot and must not be performed with human patients directly. Consequently, nonclinical experiments are indispensable to decide whether a therapeutic candidate has scientific merit for further development, leading to its evaluation in a clinical trial setting [1]. Therefore, experiments should be well designed, responsibly executed, analysed, and interpreted to generate reproducible, ethically acceptable, and scientifically reliable data that will not mislead other researchers and clinicians. Experimental designs are often compromises between two fundamentally contradictory philosophies [1]: 1) A reductionistic approach, meaning that a biological system is broken down into its smallest possible entities. Experiments focus only on specific biological aspects and benefit from being as simple as possible. These types of experiments, however, risk having limited relevance. 2) An approach to recapitulate clinical reality as far as possible, i.e., mimic the clinical disease with all its associated complexity. However, by increasing complexity, the diversity and heterogeneity of signals will increase concurrently, making it virtually impossible to dissect clear mechanisms [1]. Knowing the strengths and weaknesses of the experimental design therefore is fundamental for correct interpretation.

This is particularly important when dealing with drugs designed to combat Glioblastoma (GB), a particularly aggressive and lethal primary brain tumour. After diagnosis, only ~5% of all patients survive five years or longer [2]. With an incidence of ~3 per 100,000 persons in the United States, GB is unfortunately the most common primary brain tumour in adults and accounts for 16% of all primary brain tumours [3]. Although GB exhibits a rather low mutational burden [4,5], these tumours are characterised by pronounced intra- and intertumour heterogeneity [6], making personalised medicine approaches particularly fraught. Heterogeneity is reflected on the morphological level as well, formerly giving GB the affix ‘multiforme’ [7]. Tumours are characterised by a complex histology and particular growth pattern. Key features of GB are diffuse and unremitting growth, extensive pseudopalisading necrosis, excessive vascularization in the peripheral layers and a highly infiltrative nature [8,9,10,11]. Despite the more or less complete absence of pre-invasive GBs, extra-neural metastases occur in only 2% of all cases [9,12].

Current standard therapy for GB, referred to as the ‘Stupp protocol’, includes maximal safe surgical resection followed by radio- and chemotherapy, the latter with the alkylating agent Temozolomide (TMZ), which leads to a mean patient survival of only 15 months [13]. The relative inefficacy of therapy can be attributed to the inherent challenges GB possesses: GB cells are highly resistant to radiation or cell death-inducing substances while the tumour—if not the tumour bulk at least the invading cells—is protected from such substances by the blood brain barrier (BBB) [7,14]. The effectiveness of TMZ is believed to depend mainly on the methylation of the O^6^-position of guanine that mispairs with thymine during the subsequent DNA replication cycle. This, in turn, activates the mismatch repair (MMR) system which converts the mismatches into critical secondary pre-apoptotic lesions, ultimately leading to DNA double strand breaks and eventual apoptosis [15]. The clinical use of TMZ is, however, limited by its general toxicity, as it modifies the DNA of all cells in an indiscriminate manner. Severe cases of haematological toxicity as well as hepatotoxicity have been described in the literature [16]. Aiming to improve BBB penetration, target specificity and TMZ’s stability, various tumour-specific delivery strategies like nanoparticles have been developed over the recent years [17].

The therapeutic success of TMZ is assumed to depend on the absence of the methylguanine-DNA methyltransferase (MGMT). MGMT is a repair enzyme that removes the methyl group from the O^6^-position of guanine, thereby preventing further mismatches and preventing MMR activation. Hypermethylation of the promoter region of MGMT is found in 30–60% of all GB patients [18]. MGMT, however, is a highly debated predictive biomarker for therapy response, as the MGMT status does not universally correlate with TMZ responsiveness [19].

As the precise nature of this drug’s effect on the cells might be still not fully understood, it is important to create the correct experimental context, as TMZ is also an important component of complex combination therapies, such as the RIST (rapamycin, irinotecan, sunitinib, TMZ) protocol or the CUSP9 (coordinated undermining of survival paths with nine repurposed drugs) approach [20,21]. Importantly, in this context TMZ has been shown, even at low concentrations, to enhance the response of GB cells to radiation, or, in combination with a pharmacological PI3K/mTOR inhibitor, to break the increased apoptosis-resistance of certain GB cell populations [19,22].

In the following review, we will discuss the experimental systems that have furthered our understanding of GB and TMZ and highlight the inherent and necessary limitations of these experiments that have led to gaps in our mechanistical understanding.

## 2. Experimental Limitations 1: Finding Your Rhythm

“Alle Dinge sind Gift, und nichts ist ohne Gift; allein die Dosis machts, daß ein Ding kein Gift sei.”

*Theophrastus Bombast von Hohenheim*

When assessing the effectiveness and efficiency of a (potential) drug in a laboratory setting, it is paramount to work with concentrations and dosing schedules, which are relevant for a clinical transfer, i.e., concentrations need to be realistically achievable in the organ or tissue of interest. Results obtained with concentrations much higher than what can be achieved in patients may only be helpful to elucidate toxicities and off-target effects [23]. While it has frequently been suggested that a single high drug dose might be a good surrogate for repeated re-administration, we find this notion lacking in evidence [24]. Despite advances in in silico modelling, defining the clinically relevant and achievable concentration of a novel agent *a priori* remains virtually impossible [25]. Studying already approved drugs, however, has a clear advantage: pharmacokinetic and -dynamic studies including exposure data and toxicity findings are available—for most clinically approved drugs, but by no means all of them—and can be used to design nonclinical studies at concentrations and dosing schemes which reflect clinical reality [23].

With regards to TMZ, researchers can take advantage of over 30 years of clinical experience. In 1987, TMZ was evaluated in a phase I study for treatment of gliomas, got approval for recurrent GB and anaplastic astrocytoma in 1999, and, finally, was approved for first-line therapy of newly diagnosed GB in 2005 [13,26,27]. Many studies assessed basic pharmacokinetic parameters of TMZ like the absorption into the blood/plasma, its metabolism and excretion via urine in the elderly [28,29,30,31], while only a limited number of studies assessed the pharmacokinetics in infants and children [32], or the neuropharmacokinetics of TMZ including its penetration into the cerebrospinal fluid (CSF) or the peritumoural tissue [33,34,35].

Important pharmacokinetic key parameters that need to be considered in this context are the maximum concentration (c_max_) and the area under the curve (AUC), which integrates drug exposure over time, and is typically calculated from time zero to infinity. While c_max_ depicts drug exposure only at the time of maximum concentration, AUC allows one to depict cumulative tissue exposure in more detail as it takes bioavailability, absorption and elimination rates into account [23]. When referring to plasma drug levels, the AUC can be used to determine drug exposure in different tissues [36].

**Figure 1:**
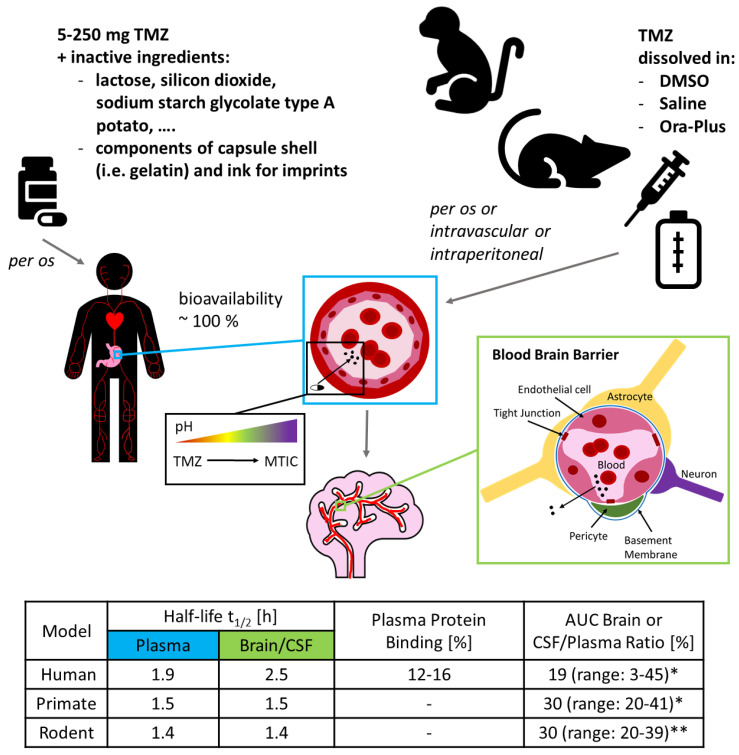
Pharmacokinetic features of Temozolomide (TMZ) and differences among species. This figure depicts key pharmacokinetic data from humans and experimental animal models. Model according to [15]. Information on clinical grade TMZ see package insert (URL: https://www.msd.de/fileadmin/files/fachinformationen/temodal_hartkapseln.pdf, link accessed on 29 May 2020). Information on TMZ for animal experiments see [20,41,42,43,44,45]. Table according to [31,33,34,35,40,46,47,48,49,50,51,52,53]. Dash) data not reported; (*) range of absolute area under the curve (AUC) brain or cerebrospinal fluid (CSF)/plasma ratio; (**) range of mean AUC brain or CSF/plasma ratio.

The pharmacokinetic features of TMZ are well known (Figure 1). TMZ can be detected quickly in the plasma after oral administration where it reaches peak levels after approximately 1.2–1.5 hours [31,33]. Several neuropharmacokinetic studies with patients suffering from brain tumours, which are summarized in a recent letter written by Stepanenko and Chekhonin, have shown that on average only 20% of systemic drug levels reach the brain (measured as mean brain interstitium or CSF AUC to plasma AUC ratio; values vary between 3.3% and 44.9% depending on the study), and that maximum concentrations of TMZ in the brain interstitium or CSF range from 1 to 10 µM [19]. While many guidelines exist on how to translate experimental conditions from in vitro studies to in vivo studies or to clinical trials, the guidelines on how to extrapolate pharmacokinetic data in the other direction are sparse [37]. Consequently, it is not surprising that different suggestions exist on how to use pharmacokinetic parameters. The c_max_ values detected in the brain interstitium were used as the basis for the TMZ concentrations applied in some in vitro studies [38,39], while others prefer to use cumulative tissue exposure concentrations calculated from AUC values [40]. As TMZ needs to be activated at physiological blood or tissue pH, it would be even more accurate to determine the concentration of its—unfortunately extremely short-lived—metabolites, such as the methyldiazonium ion. To the best of our knowledge, no primary data exists showing how much of the respective metabolites finally end up at the tumour cells in the human brain.

Although data on important pharmacokinetic parameters are available, many nonclinical studies apply TMZ concentrations that are beyond clinical relevance. Analysing PubMed-listed publications in a recent review, we found that often concentrations ranging from 100 µM up to 4000 µM are applied [15]. Considering the proposed mode of action of TMZ, one can speculate that at higher concentrations which result in greater DNA methylation and consequently, greater DNA damage, different DNA damage response pathways get activated compared to clinically relevant concentrations which are in the lower micromolar range.

In addition, cellular responses towards TMZ were shown not only to depend on the dose applied but also on the dosing scheme. Comparison of five different clinically relevant dosing schemes of TMZ in vitro revealed differences in the clonogenic survival of the GB cells depending on the dosing scheme used [39]. Even before these, Stevens and colleagues observed schedule dependency of TMZ activity in various mouse models [54], which was confirmed by Newlands and colleagues, who demonstrated schedule-dependent clinical activity of TMZ in glioma and melanoma patients in a phase I trial [29]. It becomes increasingly obvious that the dosing scheme of TMZ drastically influences the outcome [13,55,56,57,58,59]. Nevertheless, most in vitro studies still use a high single dose of TMZ as a surrogate for multiple doses.

While the pharmacodynamic details of TMZ in patients might not be fully elucidated yet, data clearly indicate that clinically relevant concentrations of TMZ are in the lower micromolar range. Therefore, every experimental design aiming to investigate cellular responses towards TMZ should use these low concentrations and a schedule that closely mimics the clinical standards.

## 3. Experimental Limitations 2: There is no Alkahest

“There is not more neutrality in the world. You either have to be part of the solution or you’re going to be part of the problem.”

*Eldridge Cleaver*

A key difference between the experimental use of TMZ and its clinical application lies in the delivery. In a therapeutic setting, TMZ is administered in the form of hard-shelled capsules, ranging from 5 to 250 mg of the drug and a significant amount of lactose as packing material. In contrast, during cell culture experiments TMZ is routinely dissolved in dimethyl sulfoxide (DMSO), an organic solvent commonly used in experimental settings for difficult to dissolve compounds, due to being miscible in water. Interestingly, for animal experiments it is frequently not stated which solvent was used to administer the drug, although DMSO [41,42], saline [20,43] and Ora-Plus [44,45] seem to be commonly used (see Figure 1).

DMSO, however, is not an inert solvent. While the clinical application of DMSO was initially stopped—possibly prematurely—in 1965 due to fears of off-target effects [60], it was eventually approved for clinical use by the United States Food and Drug Administration in 1978 [61]. DMSO has shown efficacy in the treatment of, among others, gastrointestinal, dermatologic and rheumatologic disorders, as well as chronic prostatitis, amyloidosis, and traumatic brain oedema [61]. The impressive list of reported systemic side effects, such as nausea, vomiting, diarrhoea, haemolysis, rashes, renal failure, hypertension, bradycardia, heart block, pulmonary oedema, cardiac arrest, and bronchospasm [61] leaves little doubt of DMSO’s low-dose toxicity [62,63]. This, in turn, means that any analysis of TMZ dissolved in DMSO is in essence the analysis of a combination treatment of two toxic substances, both of which potentially induce apoptosis [15,64,65].

**Figure 2:**
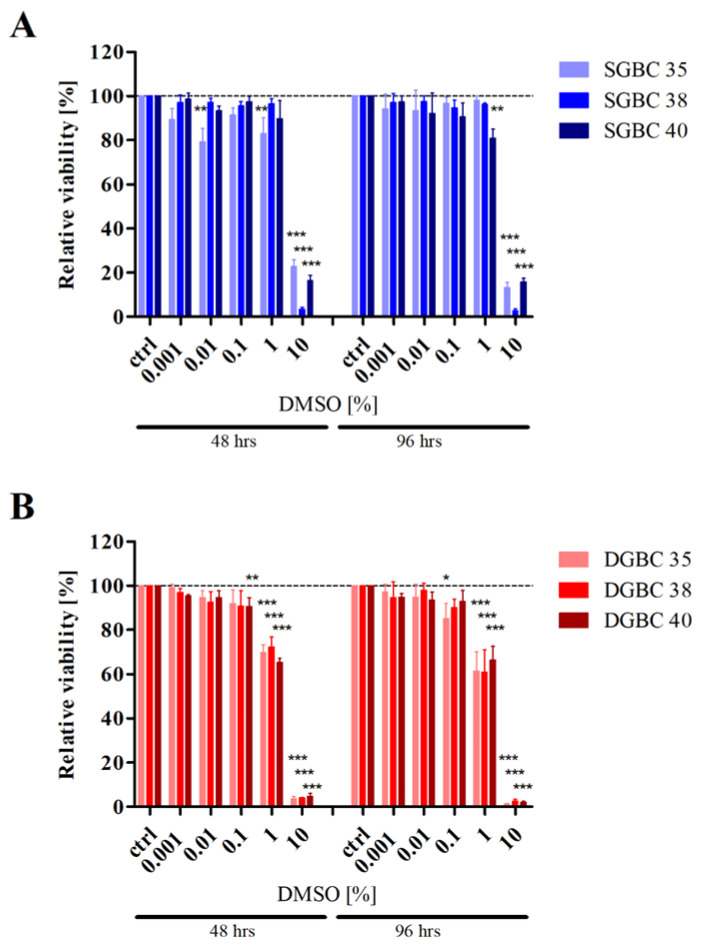
Effect of the solvent Dimethyl sulfoxide (DMSO) on metabolic activity of glioblastoma stem cell-like cells and their differentiated progeny. (**A**) Glioblastoma stem cell-like cells (SGBCs) and (**B**) their differentiated progeny (DGBCs) from three individual patients (35, 38 and 40) were stimulated with indicated percentages *v*/*v* of the solvent DMSO for 48 and 96 hours. The relative viability of the respective populations was normalized to untreated control populations (ctrl). The columns represent the mean and positive standard deviation of three independent experiments carried out in triplicates. Cell culture conditions and experimental setup were as previously described [70,74], statistical analysis was performed using a one-way ANOVA test, followed by the Tukey’s multiple comparison test; *, *p* ≤ 0.05; **, *p* ≤ 0.01; ***, *p* ≤ 0.001.

Furthermore, the use of physiologically too high TMZ concentrations in in vitro experiments always correlates with increasing concentrations of DMSO. Depending on the specific chemical vendor, the suggested solubility of TMZ in DMSO varies between 9.7 mg/mL and 39 mg/mL, corresponding to a stock concentration of 50–200 mM (Table 1). In in vitro studies, where extremely high concentrations of TMZ (1000–4000 µM) are applied, the DMSO content corresponds to a minimum of 0.5–2% *v*/*v*, when using the highest recommended stock concentrations of 200 mM. Such concentrations of DMSO can have a whole range of effects on various cellular systems, as highlighted in Table 2. Importantly, the major cellular compound of the brain, astrocytes, which comprise ~50% of all brain cells [66] and are the putative progenitors of GB [67], is also particularly susceptible to the effects of DMSO. One study shows that DMSO concentrations of 0.5% and 1% enhance proliferation and GFAP (glial fibrillary acidic protein, an astrocyte marker) expression, while concentrations of at least 5% are needed to reduce astrocyte survival and GFAP expression [68]. While another study suggests that in astrocytes 1% DMSO is sufficient to decrease both, viability and expression of glutamate transporters, a typical sign for oxidative stress [69]. In line with this finding, the mitochondrial membrane potential was decreased, reactive oxygen species production was increased, and cytochrome c was released into the cytoplasm, ultimately leading to the activation of apoptotic proteins, such as caspase 3 [69]. Neurons, the basic functional units of the nervous system including the brain, are even more sensitive to DMSO; here, already 0.5% and 1% are sufficient to cause a profound loss of viability within a relatively short time span [64,68]. Our own work shows that DMSO also affects cultured GB cells at relatively low concentrations, i.e., at concentrations necessary for treatment with physiologically relevant amounts of TMZ. When using primary-cultured stem cell-like cells (SGBC) and their short-term differentiated counterparts (DGBC) [70], which mimic the tumour much more closely than established cell lines [71,72], we could show a significant reduction in viability (Figure 2). The stem cell-like cells, which have been proposed to exhibit increased resistance to therapy [73], are, consistent with this current literature, more resistant to DMSO than differentiated cells.

Therefore, every experimental finding regarding the effects of TMZ on cells, particularly tumour cells such as GB, which are derived from cells sensitive to DMSO, must always be considered with the role of the solvent in mind. This conclusion not only pertains to therapeutic, but to mechanistic studies also. DMSO has been shown to cross the BBB [83] and, of course, is a solubiliser, affecting the cellular uptake of drugs [84]. Therefore, the pharmacokinetics and -dynamics of TMZ and its active compounds will certainly profoundly differ between the experimental conditions and the clinical reality.

## 4. Experimental Limitations 3: GB’s Next Top Model

“… der denkende treue Beobachter lernt immer mehr seine Beschränkung kennen, er sieht: je weiter sich das Wissen ausbreitet, desto mehr Probleme kommen zum Vorschein.”

*Johann Wolfgang von Goethe*

There are several in vitro approaches to test the effect of TMZ on GB. While we do not attempt to provide a detailed overview on all the models that have ever been developed (these are extensively described elsewhere, for example [1,85,86]), we will highlight the limitations of the most commonly used ones.

### 4.1. ‘Classic’ GB Cell Lines

Established cell line models have the undeniable advantage of being reductionist and highly amenable, thereby allowing one efficiently to dissect cellular mechanisms. They are easy to handle and can be readily expanded for an unlimited amount of passages in vitro, thus, providing enough cells for experimental use. In addition, cell line-based models are cost effective and reproducible. The most commonly used GB cell lines that are commercially available are summarized in Table 3. The first GB cell lines were established throughout the 1970s and 1980s out of patient biopsies that were passaged in serum-supplemented media [85] and today a whole range of genetically distinct options is available.

GB cell lines have been widely used to study the biology of GB and to test therapeutic approaches, including elucidating the effects of TMZ. During recent decades, however, a growing body of evidence suggests that GB cell lines are poor representatives of the primary tumour isolated from patients in terms of genetics, mRNA profiles and protein expression [71,87,88]. These differences can be attributed to the culture conditions, as classic cell culture exerts an in vitro selection pressure for proliferative capacity resulting in cellular homogeneity [87,88]. While all GB cells must compete for growth factors, metabolites and oxygen in vivo, cell culture conditions are optimized for highly proliferative GB cells [87].Cultivation in serum-supplemented media (Table 3), for instance, is associated with astrocytic differentiation by which the genotypic and phenotypic variety found in patient-derived GBs gets lost [71,89]. Furthermore, these cells are typically grown adherently as a monolayer on plastic. This way of cultivation ignores the three-dimensional growth that a tumour normally portrays and the contact to, as well as influence of the tumour microenvironment (TME), such as additional cell types and blood-delivered secretable factors. Therefore, in vivo models in the form of orthotopic xenografts are a possibility to get a more realistic view. Nevertheless, establishing a reliable mouse model based on classic GB cell lines is challenging—if transplantation is successful, which is difficult for some cell lines (Table 3), some tumours hardly recapitulate important hallmarks of GBs, i.e., its heterogeneity or infiltrative behaviour [90,91]. Yet, most of our knowledge regarding the effects of TMZ on GB are derived from works with these cellular systems.

### 4.2. Primary Cultured, Patient-Derived GB Stem Cell-Like Cells and Their Differentiated Progeny

Culturing primary patient material as spheroids (SGBC) in neurobasal medium recapitulates the genotype and in vivo tumour biology more precisely than established cell lines. Unlike in GB cell lines, gene expression patterns were preserved in SGBCs even after long-term passaging [71]. The addition of serum, however, induces the differentiation of those cells (DGBC) which then grow adhesive as monolayer, and passaging leads to great changes in their genomic profile [71,72,127]. Intriguingly, some SGBCs escape serum-induced differentiation, become more aggressive in vivo and switch to a mesenchymal phenotype [128]. Importantly, human SGBCs and DGBCs are highly tumourigenic in immunocompromised mice and give rise to lethal brain tumours characterised by cellular pleomorphism, extensive and diffuse invasion, increased vascularity, and pseudopalisading necrosis [70,127,129,130,131].

Patient-derived SGBCs are a powerful tool with clinical relevance. Several studies have shown that the success of neurosphere formation out of primary patient material is associated with the clinical outcome of the patient and can be used to predict treatment response [132,133,134]. The successful establishment of SGBC cultures is associated with shorter median progression-free as well as overall survival [134]. In addition, the time required for establishment of SGBC culture showed similar correlations—the shorter the expansion time, the worse the prognosis [134]. Follow up studies confirmed these findings [132,133,135] as well as for paediatric glial tumours [136] and could further show that the sensitivity of SGBCs to radiation and TMZ was associated with patient survival [132]. In addition, the capacity of forming a xenograft tumour in animal models and the invasiveness of orthotopically transplanted SGBCs were correlated with poorer clinical prognosis [137].

Early passages of DGBCs and their SGBC counterparts are, therefore, reliable model systems that mimic cellular heterogeneity as well as lineage hierarchy in GB and can be used to examine the different types of cells present in a tumour, identify novel tumour relevant genes and can be used for drug screenings [70,138,139].

### 4.3. Genetically Identical Cells Responding Differently to TMZ

The Cancer Stem Cell (CSC) hypothesis proposes that tumours consist of hierarchically arranged populations of cells with CSCs being at the apex of the hierarchy–a subpopulation that has the ability to self-renew and to differentiate thereby recapitulating the entire functional diversity present within the original tumour [140]. SGBC are presumed to be similar to CSC, as they show increased positivity for stem cell markers [71], but whether they represent an enrichment, a subset or what exactly the relationship between those two terms is, remains open for debate. The inevitable re-emergence of GB after surgical resection, radiation and chemotherapy suggests that within GB a subpopulation exists that is resistant to these therapies and based on the stem cell model, CSCs are often proposed to be the underlying cause [141]. Since the establishment of neurosphere culture, however, a vast amount of studies has been conducted providing somewhat controversial results.

Although great differences in the expression of proteins mediating survival and cell death can be observed in SGBCs and DGBCs, as well as displaying substantial differences in the growth rates, both populations in our hands died at comparable rates after exposure to TMZ—cell death, however, could be described as rather moderate in general [70]. Surprisingly, metabolism and cell numbers were affected to a greater extent in SGBCs upon TMZ treatment than in DGBCs [74]. In line with these reports, CD133^+^ SGBCs stopped proliferating upon TMZ treatment and got selectively depleted without induction of considerable cell death in vitro [38,142]. However, in contrast to these reports on the susceptibility of SGBCs, it has been shown that SGBCs are more resistant to TMZ and radiation than their differentiated counterparts or classic GB cell lines cultured in the presence of serum, and treatment led to enrichment of CD133^+^ cells in vivo [132,143,144,145,146,147]. Therapy resistance was associated with higher expression of drug efflux pumps and DNA repair proteins [143,147]. In addition, their self-renewal capacity seemed not to be inhibited in the presence of TMZ, neither in vitro nor in vivo [142,148,149], which is why SGBCs are also considered to be key players in driving tumour relapse. Another layer of complexity is added to the whole story, as TMZ treatment was shown to increase the stem cell-like population by interconversion of differentiated glioma cells [70,144].

Primary data on TMZ-induced effects in SGBCs and DGBCs should be interpreted with care. Finding a consensus is complicated as different TMZ concentrations and schedules are used in these reports. In addition, caution has to be taken, as some studies employed cells initially cultured in serum-supplemented medium [146,147,150] and often stem cell properties have not been investigated in detail. In general, controversies largely stem from an only vague definition of what GB stem cells are and the lack of specific markers defining SGBCs and adequate methodologies to investigate SGBCs in vitro, i.e., their stem cell properties. Moreover, while the discovery of SGBC/DGBC cultivation was a huge improvement compared to studies performed on GB cell lines, they are still lacking an important determinant of the pathologic course of GB—the TME. The TME is constituted by a variety of cells, i.e., astrocytes, fibroblasts, neurons, microglia and endothelial cells [151] but also infiltrating peripheral immune cells are found within tumour tissue [152,153]. It is further shaped by soluble signal molecules, receptor ligands and extracellular vesicles as well as by a unique extracellular matrix (ECM) and specific conditions like hypoxia and acidosis [14]. Consequently, when aiming to understand GB biology and the effectiveness of any therapy, the TME should be included in the investigation sooner or later as well.

### 4.4. Co-Culture Systems Mimicking the TME

The simplest way to mimic the TME is the principle of co-culturing different cells together in an in vitro manner. Nowadays multiple ways of co-culturing have been described ranging from simple approaches like just mixing two cell populations together to more complex settings such as using transwell systems, solid support systems in which cells are cultivated on porous scaffolds or gels and microfluidic systems, in which cell populations are separated by fluid channels or membranes [154,155]. Recently, Heinrich and colleagues established so-called three-dimensional bioprinted mini-brains in which mouse glioblastoma-associated macrophages and mouse GB cells are co-cultured to test cell-interactions following the use of therapeutics. Their proof-of-concept study possibly paved a way to more complex multicellular TME mimicking systems [156]. The advantage of co-culture systems is, of course, that they are a more representative human-like tissue model than single cell culture. Furthermore, in comparison to animal models, they are simple enough to study specific mechanisms and can be performed on a much higher throughput.

It was deduced from co-culture experiments that the interaction of GB cells with astrocytes, whose inherent function is to protect the brain, provides increased resistance towards several types of chemotherapy, including TMZ [157,158]. This effect was dependent on cell to cell contact and mediated by gap junctional communication [157,159]. In addition, TMZ treatment itself promoted gap junctional communication via the induction of connexin 43 on tumour cells [160]. Interestingly, untransformed human astrocytes seem to be resistant to physiologically relevant concentrations of TMZ in vitro. Cell viability was only slightly decreased, while no cell cycle alterations or cell death were observed in the presence of TMZ [111,157,161,162]. Primary microglia cultures as well as tumour-associated or ‘normal’ endothelial cells seem to be resistant towards TMZ treatment, too [163,164]. Microglial cells have been associated with stimulating proliferation and invasion of GB cells, as well as mediating resistance towards cytotoxic drugs, such as vincristine and TMZ [165]. Co-culturing of GB cells with endothelial cells as well as stromal cells, which are important constituents of the perivascular niche of the tumour [166], led to upregulation of genes involved in angiogenesis, resulted in enhanced PI3K/Akt and Ras/MAPK signalling in GB cells and increased their resistance towards TMZ treatment [167]. In summary, primary data supports a resistance mediating role of the TME which is likely to contribute to a rather low five-year overall survival of less than 10% [168].

Co-culture systems have helped to study the TME and its role in tumourigenesis and therapy response in more detail. However, as monoculture systems, they suffer from the general limitations cell culture possesses—the complexity of the disease and its interactions with the living host cannot be replicated in a petri dish.

## 5. Experimental Limitations 4: Of Mice and Men

“The best-laid schemes o’ mice an’ men // Gang aft agley”

*Robert Burns*

Whole-animal models undoubtedly provide a promising option when aiming to evaluate the potential of therapeutic strategies. The complex interplay between tumour and host can be modelled only in vivo. Important determinants of the host are not only the unique TME but also anatomical barriers, such as the BBB. Most commonly, rodents, i.e., mice or rats, have been used to investigate GB biology and therapy response in vivo. These models can be divided into three categories: (1) chemical carcinogen-induced models, (2) genetically engineered models (GEMs), and (3) xenograft models.

Experimental tumours are typically induced in rodents by local, oral, intravenous or transplacental exposure to carcinogens, such as N-ethyl-nitrosurea (ENU) or methylnitrosourea (MNU) [85]. However, none of the commonly used chemicals has ever been implicated in human GB development and further, not surprisingly, the experimental brain tumours thus created have histological, pathological, and genetic features distinct from those of humans [85,169].

GEMs are of greater relevance. The development of genome editing technologies such as viral vectors or CRISPR tools in combination with conditional gene expression technologies (time and/or tissue specific, i.e., CreLoxP strategy) promoted the development of GEMs [1]. In most GEMs key signalling molecules known to be deregulated in GB such as PI3K, EGFR, TP53, Rb, Ras, CDKN2A are modulated [85,170]. While GEMs are closer to human GBs in terms of histopathology, they cannot *per se* recapitulate the great genomic and phenotypic heterogeneity characteristic of GB. Furthermore, tumour initiation cannot be controlled in this type of model, which is why therapeutic studies are difficult to perform [85].

In contrast, xenograft models are an excellent tool to address this question. These tumours are usually established from classical GB cell lines or from primary patient material cultivated as neurospheres. Furthermore, with the aim of bypassing any divergence induced by cell culture processes, patient-derived xenografts, also called ‘Avatar models’, have been developed. Patients’ biopsies are directly transplanted into immunocompromised mice, thereby conserving intratumoural heterogeneity [171]. The grafts can be either heterotopic or orthotopic. Heterotopic xenografts are most commonly subcutaneous into the flanks, i.e., a region that does not resemble the unique microenvironment of the brain at all. Furthermore, these models lack an important anatomical barrier—the BBB, which limits the access of many drugs to the tumour. The most realistic models, therefore, are orthotopic xenografts where tumour cells are transplanted directly into mouse brain by free-hand procedure or stereotactic surgery [169]. However, a drawback of this model is that the transplantation procedure itself creates an injury by which normal tissue architecture and physiology are disrupted [1].

While the efficacy of TMZ was already evaluated in preclinical mouse models for other solid tumours like melanoma, Lewis lung carcinoma, colon carcinoma, and ovarian sarcoma in 1987 [54], animal data on TMZ for glioma were only published in 1994 [125]. Surprisingly, TMZ was first tested in glioma patients in a non-randomised phase I study at the Charing Cross Hospital in 1987 [29,172,173]. By 2000, recruitment for phase II and III studies began, although only seven relevant preclinical studies had been published by that time [174]. From today’s perspective, the basis for the decision to conduct and proceed with clinical trials on TMZ in glioma patients is not clear [174]. Nevertheless, studies on glioma mouse models could show that TMZ significantly improved the outcome. A meta-analysis on the efficacy of TMZ in preclinical animal models performed by Hirst and colleagues revealed that TMZ-treated mice survived twice as long as the control group (median survival ratio: 1.88) and tumour volume was reduced by approximately 50% [174]. Furthermore, preclinical animal data support the schedule and dose dependent effect of TMZ [174].

Rodents are no doubt useful tools with which to study tumour development and drug response, but, as with every approximation, one has to be acutely aware of the model’s limitations, i.e., differences in drug absorption, distribution, metabolism, and excretion (ADME) (Figure 1) to name but a few [175]. For instance, although TMZ is one of the few chemotherapeutics that can cross the BBB, only about 20% of the plasma concentration is detected in the human CSF [33,34]. In contrast, some studies have shown that up to 30% of the plasma concentration can be detected in the brain of rats and the CSF of rhesus monkeys respectively [40,51]. These contrasts might be in part attributed to differences in the BBB, as huge variations in the expression and substrate specificity of BBB-permeability related proteins, such as transporters, receptors, and tight junction proteins have been shown to exist, on the one hand between normal human brain and GB microvessels, and on the other hand between humans, monkeys and rodents, but also even within different rodents [176,177,178]. Taking into account the huge differences, rodents may not be the optimal model for investigating the BBB and species distinctions in general may limit the transfer to humans. Species distinctions might also be the reason why in a few mouse models TMZ greatly prolonged survival of mice bearing GB cell line or SGBC/DGBC grafts [126,179,180], while in humans, the addition of TMZ prolonged median overall survival by only 2.1 months [13]. When talking about species-differences in ADME properties, it is also important to mention the most important drug-metabolising enzymes, cytochromes P450 (CYPs450). Interestingly, the tissue expression patterns as well as the catalytical activity and specificity of CYPs450 vary greatly among different species, but also among humans, where they are considered as a major source of variability in drug pharmacokinetics [15,181,182]. According to our current understanding, however, CYPs450 do not influence the metabolism of TMZ as suggested by the following experimental findings: the activation of TMZ is independent of the presence or absence of liver microsomes, the mean half-life is comparable between aqueous buffers and human patients and interpatient variability of TMZ pharmacokinetics is low [31,34,183,184].

## 6. Experimental Limitations in the Context of TMZ’s Effect on the Immune System

In recent years, our understanding of how TMZ affects the cells has shifted. A role for TMZ in autophagy and senescence has been identified [185], while its role in protein methylation is still underexplored [15]. However, the experimental limitations to study TMZ are nowhere more apparent than when exploring its effect on the immune system.

The lack of a functional immune system required to allow successful transplantation of human cells to other species is a further inherent drawback of xenograft animal models. Much of the knowledge about the effects TMZ has on the immune system, therefore, has been derived directly from GB patients or from syngeneic immunocompetent rodent models. Syngeneic rodent models are generated by grafting stable rodent cell lines, of which most of them have been generated by exposing rodents to chemicals like ENU or MNU (with all the inherent limitations of established cell lines already discussed), into the respective immunocompetent host [186,187].

Chemotherapy-induced lymphopenia, which is defined by a decreased density of lymphocytes in the blood, is a common adverse event in the treatment of cancer [188,189]. TMZ administered systemically also has immunosuppressive side effects. The treatment with TMZ can cause lymphopenia and T cell dysfunction in patients resulting in an increased risk of viral and bacterial infection. In such cases, it is indicated that patients should be monitored for opportunistic infections [190]. Furthermore, it was demonstrated that concomitant standard radiation and TMZ (RT/TMZ) provokes a strong reduction in lymphocytes but numbers of lymphocytes slowly come back to normal when treatment with TMZ is stopped [191]. Another study showed that the frequency and absolute numbers of natural killer (NK) cells, which are known to destroy tumour cells by their ability to initiate apoptosis in target cells with reduced levels of MHC molecules (‘missing-self’ recognition), are decreased in RT/TMZ-treated GB patients [189]. Therefore, immunosuppression by TMZ is also a challenge for effective anti-tumour immunotherapy gaining currently great attention in GB.

GB itself has developed many different strategies to evade the immune system and is highly immunosuppressive, locally as well as systemically [192,193]. The TME for instance is highly immunosuppressive by recruitment of T regulatory cells (Tregs). Tregs are responsible for immune homeostasis and maintaining immunity but can also suppress a tumour-specific immune response by upregulation of immune-checkpoint molecules, secretion of inhibitory cytokines and via metabolic pathways [194]. It is not particularly surprising to use Treg depleting strategies in order to improve an anticancer immune response. In this regard, it is remarkable that low dose of TMZ depletes the number of circulating Tregs in a TMZ-resistant rat model of glioma [194], suppresses the frequency of Tregs in an intracranial GL26 glioma animal model [195], and after RT/TMZ treatment the proportion of Tregs is found to be decreased in peripheral blood from high grade astrocytoma patients [196]. On the other hand, it was demonstrated that the frequency of Treg cells increases after RT/TMZ therapy in GB (malignant glioma) [197]. As a matter of fact, the right timing and dosing of treatment are crucial for the effect of TMZ on Tregs [198]. Tregs are not the only immune cells of the inhibitory cellular network, as suppressive tumour-associated neutrophils, glioma-associated microglia/ macrophages (GAMs), and myeloid-derived suppressor cells (MDSCs) are critical for anti-tumour therapies, too. MDSCs are a heterogeneous population of immature myeloid cells. GB secretes, for instance, chemokine C-C ligand 2 (CCL2) to attract monocytes to migrate to the TME and converts monocytes, among others, to MDSCs [199]. Thesein turn can convert naive T cells into induced Tregs [200]. MDSCs and GAMs are the majority of infiltrating immune cells and might be central in immune suppression in the TME [193,201,202]. Indeed, increased counts of MDSCs in recurrent GB indicate a poor prognosis of the disease, in contrast to reduced levels of MDSCs in GB patients which correlate with an extended survival rate [203]. In the case of MDSCs, immunodepletion could be advantageous as well, but so far, it is not completely clear whether and how TMZ interferes with MDSCs. Ex vivo, TMZ provokes the intrinsic mitochondrial pathway of apoptosis in human myeloid cells and the combination of TMZ with a CCL2 antibody, which blocks recruitment and polarization to MDSCs, improved survival greatly in a mouse model [199,204]. Whether these findings can be recapitulated with human GB patients remains to be investigated.

Systemic administration of TMZ results in lymphopenia and can limit the efficacy of immunotherapies; however, immunodepletion might be beneficial when Tregs, MDSCs or other cells involved in immunosuppression in the GB environment are depleted [204]. Combining TMZ and immunotherapy may be successful but will be a delicate balance of timing, dosing, and local rather than systemic administration of TMZ [192].

## 7. Conclusions and Outlook

Here, we have identified four major limiting factors that impede a better understanding of TMZ’s complex role in the treatment of GB. The first two limitations identified can be easily overcome. There is no inherent reason why recapitulating clinical dosing and scheduling in experimental systems cannot be applied, at least in cell culture models. In both, in vitro and in vivo experiments alternatives to DMSO as a solvent are readily available. Indeed, as soon as experiments are performed with physiologically achievable concentrations of TMZ, water is a perfectly adequate solvent for TMZ, allowing for a stock solution of 1 mM, versus a stock solution of 50–200 mM when using DMSO. Therefore, these limitations should be a purely historical problem, i.e., any information on the function of TMZ gained from experiments using too high concentrations (as surrogate for multiple application, or just because physiological concentrations showed no effect) and DMSO as a solvent, should be viewed as potentially compromised.

The second set of limitations identified are also of little surprise. After all, every model simplifies and therefore, recapitulates imperfectly. Moreover, while this also suggests that caution should be applied when interpreting data obtained from these models, an opportunity presents itself here for further improvements.

A promising option to mimic human GB is the use of GB organoids (GBOs) [205]. These GBOs are obtained from patients generated by excising tissue along the tumour margin with minimal necrosis and without cauterization. The obtained GBOs are cultured in specific medium without exogenous use of epidermal growth factor, fibroblast growth factor, serum and no exogenous extracellular matrix to maintain their cell-cell interactions, subtypes and gene expressions of the parental tumour. Intriguingly, these GBOs also contain non-neoplastic cells like microglia, T cells, stromal cells, and oligodendrocytes. Additionally, when transplanted orthotopically into mice, the GBOs engrafted efficiently, displayed signs of aggressive infiltration and when grown to a sufficient size, even had a hypoxic core comparable to GB in humans. Furthermore, Jacob and his group of co-workers have described their method as being readily transplantable into murine models within one-month post-surgery, enabling the testing of personalized medicine [205].

The downsides of available rodent models have fuelled the search for GB’s next top model. One approach to adapt the TME is, e.g., the use of humanized mice models by engrafting immunodeficient mice with functional human cells and tissues [206,207]. In recent years, larger animals as pigs or dogs have gained interest, too. Porcine GB models are characterised by an anatomically more relatable gyrencephalic structure and a more similar BBB physiology to humans than murine models [208]. Furthermore, canine models, as presented by Koehler and colleagues, have been described to be a translationally relevant model to humans due to the spontaneous occurrence of gliomas in dogs and therefore, the naturally existing TME, the presence of intratumoural heterogeneity and an intact immune system [209].

After the rather limited successes of personalised medicine approaches [210,211], the focus has in recent years shifted towards immunotherapy, an approach which in many aspects is the complete opposite of personalised medicine. While intriguing data exist that show an effect of TMZ on the immune system, most currently available model systems are not suited to address this issue adequately. This is of particular importance, as immunotherapy is becoming an important aspect of future treatment options for GB [212], yet, as discussed, the presence of TMZ might limit the efficacy of this treatment approach. However, it has also been suggested that senescence, which can also be induced by TMZ [185], can provide additional targets for immune therapy [213]. To resolve this conflict appears to us to be the most pressing issue regarding our understanding of TMZ.

## Figures and Tables

**Table 1:** Solubility of Temozolomide in dimethyl sulfoxide (DMSO). Online references were accessed on 24 July 2019.

Vendor	Solubility in DMSO [mg/mL]	Stock Concentration [mM]	Online Reference
BioCrick	9.7	50	https://www.biocrick.com/Temozolomide-BCC4386.html
TargetMol	9.7	50	https://www.targetmol.com/compound/Temozolomide
Tocris	9.7	50	https://www.tocris.com/products/temozolomide_2706
Abmole	>20	>100	http://www.abmole.com/products/temozolomide.html
Merck/Sigma Aldrich	>20	>100	https://www.sigmaaldrich.com/catalog/product/sigma/t2577?lang=de&region=DE
AbaChem Scene	20.83	107.29	https://www.chemscene.com/85622-93-1.html
MedChem Express	20.83	107.29	https://www.medchemexpress.com/Temozolomide.html
Apex Biotechnology	>29.61	>152.6	http://www.apexbt.com/temozolomide.html
Selleckchem	38	195.72	https://www.selleckchem.com/products/Methazolastone.html
Adooq	39	200.87	https://www.adooq.com/methazolastone.html
Santa Cruz Biotechnology	39	200.87	https://www.scbt.com/scbt/product/temozolomide-85622-93-1

**Table 2:** Toxicity of Dimethyl sulfoxide (DMSO) in various cellular systems.

DMSO Concen-Tration Used (*v/v*)	Cellular System	Reported Effects	Additional Comments	Reference
0.1, 0.5, 1, 1.5, 2, 3 and 5%	Human umbilical vein endothelial cells, RAW264.7 mouse macrophage cell line, MCF-7 human breast cancer cell line	Growth inhibition at concentration > 0.5%	Only looked at cell viability	[75]
2, 4, 10, 20, 50 and 100%	Caco-2 cell line (enterocyte-like)	Lactate dehydrogenase release and Neutral Red uptake significantly altered at concentrations > 10%	Looked at alterations in apical membrane permeability or on cell-to-cell tight junctional complexes	[76]
0.001, 0.01, 0.1, 1, 3, 5, 7 and 9%	GSF3.2 goat skin fibroblast cell line	0.01–0.001% enhance proliferation, 0.5–3% retarded proliferation, > 3% no cell survival	Only looked at cell viability	[77]
0.1, 0.2, 0.5, 1, 2 and 5%	Molt-4, Jurkat, U937 and THP1 leukaemic cell lines	Cytotoxic effects at concentrations > 1%		[78]
0.5, 1, 2, 2.5, 5, 10 and 20%	Peripheral blood lymphocytes	1% and 2% reduce relative proliferation, 5% and 10% reduce percentage total lymphocytes and cytokine producers		[79]
0.01, 0.1 and 1%	Embryonic stem cells	All tested concentrations lead to reduced viability, alter morphology and adhesion and lead to abnormal differentiation		[80]
1%	Human lens epithelial cells	Decreased cell viability, increased cellular apoptosis, and upregulated Bax in these cells		[81]
0.2, 0.4 and 0.6%	EAhy926 umbilical vascular endothelial cell line, red blood cells	Concentrations of > 0.2% consistently cause haemolysis in red blood cells, concentrations of >0.2% consistently cause cell cycle arrest and all concentration increase apoptosis in EAhy926	The authors conclude that DMSO is toxic to the haematologic system	[82]
0.25, 0.5, 1, 2, 3, 4, 5 and 6%	Platelets	Aggregation induced by various factors was consistently inhibited by concentrations of > 1%		

**Table 3:** Characteristics of a panel of 12 commercially available GB cell lines.

Cell Line	Age [yr]	Sex	Cell Culture Condition	Karyotype	MGMT Status (Promotor Methylation)	TP53 Status *	PTEN Status *	CDKN2A (p14ARF/p16^INK4a^) Status *	Orthotopic Xenograft Model
A172	53 ^A,B^	M ^A,B^	Adherent, Serum 10% ^A,B^	Diploid to Hyper-triploid (*n* ~ 80) ^B,D^	Negativ (methylated) ^G,H,I,J,K,L^	wt ^C,R^	Homozygous Deletion ^A,C,R^	Homozygous Deletion ^A,C,R^	T ^U,V^ NT ^W^
DBTRG-05MG	59 ^A,B^	F ^A,B^		Hypotetraploid (*n* ~ 87–91) ^A,B^	Positive (unmethylated) ^M^ vs. Negative ^J^	wt ^R,S^	Homozygous Deletion ^R^	Homozygous Deletion ^R^	T ^X,Y^
LN18	65 ^A^	M ^A^	Adherent, Serum 5–10% ^A,C^	*n* ~ 70–80, modal: 78 ^E^	Positive (un-methylated) ^G,H,I,J,L,N^	Homozygous * vs. Heterozygous ^C,R^ Missense Mutation (Cys→Ser) ^A^	Wt ^A,C,R^	Homozygous Deletion ^A,C,R^	T ^W,Z^
LN229	60 ^A^	F ^A^		Hypertriploid (*n* ~ 82) ^F^	Negative (methylated) ^F,G,I,J,K,L^	Homozygous * vs. Heterozygous ^C,R^ Missense Mutation (Pro→Leu) ^A^	wt^A,C,F,R^	Homozygous Deletion ^A,C,R^	T ^W,Aa^
LN308 (LNZ308)	65 ^C^	M ^C^		Hypertriploid (*n* ~ 75–81) ^F^	Negative (methylated) ^F,G,I,O^	null/null^C^	Splice-site (Deletion exon6) ^C,F^	Wt ^C^	T ^Bb,Cc^
M059K/J	33 ^A^	M ^A^	Adherent, Serum 10% ^A,B^	M059K: *n* ~ 65–79, modal: 75 ^A^	Positive ^N^ vs. Negative ^J^	Homozygous Missense Mutation (Glu→Lys) ^T^	Heterozygous Frameshift Deletion ^R^	M059J: wt ^R^	na
T98G	61 ^A,B^	M ^A,B^		Hyperpentaploid (*n* ~ 128–132) ^A,D^	Positive (unmehtylated + methylated) ^G,H,I,J,L,M,N,O,P^	Homozygous Misssense Mutation (Met→Ile) ^C,R^	Homozygous Missense Mutation (Leu→Arg) ^C,R^	Homozygous Deletion ^C,R^	T ^V,Dd,Ee^ NT^W^
U87 **	44 ^B,C^	F ^B,C^		Hypodiploid (*n* ~ 44, 48%) ^A,D,F^	Negative (methylated) ^F, G, H, K, L, M, O, P, Q^	wt ^C,R^	Homozygous Splice Site (c.209+1G>T) ^A,C,F,R^	Homozygous Deletion ^A,C,R^	T ^V,W,Ee, Ff, Gg^
U118MG **	50 ^A^	M ^A^		Cytogenetically similar to U138 ^A,D^	Positive (un-methylated) ^K,L,N,Q^	Homozygous Missense Mutation (Arg→Gln) ^C^	Homozygous Splice Site (c. 1026+1G>T) ^A,C^	Homozygous Deletion ^A,C^	T ^V,Hh^ NT ^W^
U138MG **	47 ^A^	M ^A^	Adherent, Serum 10% ^A,B^	Hyperdiploid to Pentaploid ^A,D^	Positive (un-mehtylated) ^H,K,L.M,O^ vs. Negative ^G^	Homozygous Missense Mutation (Arg→Gln) ^C,R^	Homozygous Splice Site ^C,R^	Homozygous Deletion ^C,R^	T ^Ii^ NT ^V^
U251 **	75 ^C^	M ^C^		Diploid (*n* ~ 46) ^B^	Negative (methylated) ^G,H,J,L,M^	Homozygous Missense Mutation (Arg→His) ^C^	Heterozygous Frame-shift Insertion (c.722_723dup) ^C^	Homozygous Deletion ^C^	T ^Ff,Jj^
U373 **	61 ^C^	M ^C^		Diploid (*n* ~ 46) ^B,D^	Low positive^H^ vs. Negative (methylated) ^G,L,P^	Homozygous Missense Mutation (Arg→His) ^C^	Frameshift Mutation ^C^	wt vs. p14 ^ARF^ Deletion Variant ^C^	T ^V,Ee,Kk^

CDKN2A: cyclin-dependent kinase inhibitor 2A gene locus; F: Female, GB: Glioblastoma, na: not available, M: Male, MGMT: methylguanine-DNA methyltransferase; n: number; NT: not tumourigenic, p14^ARF^: alternate reading frame protein of CDKN2A gene locus; p16^INK4a^: cyclin dependent kinase inhibitor 2A; PTEN: phosphatase and tensin homolog; T: tumourigenic, TP53: tumour suppressor p53, wt: wild type; yr: year. * TP53, PTEN, and CDKN2A status were verified using the COSMIC database (https://cancer.sanger.ac.uk/cell_lines) as well as the Cancer Cell Line Encyclopaedia database (https://portals.broadinstitute.org/ccle). ** Note: the identity of various GB cell lines has been questioned. Allen and colleagues could show that the widely used U87 cell line purchased from The American Type Culture Collection (ATCC) differs genetically and transcriptionally from the U87 cells from Uppsala. Nevertheless, despite lacking patient information, it is proven that ATCC derived U87 cells originated from a central nervous system tumour [92]. Furthermore, ATCC has reported that U118 and U138 cells, although derived from different individuals, have similar DNA profiles, are very similar cytogenetically and share at least six derivative marker chromosomes [A], indicating that cross contamination has occurred [92]. In addition, ATCC as well as the European Collection of Authenticated Cell Cultures have reported that their U373 stock differs from the originator’s stock and the stock held as U373 was found to be identical by short tandem repeat profiling to U251 [A,B]. Based on: (A) https://www.lgcstandards-atcc.org/; (B) https://www.phe-culturecollections.org.uk/; (C) [93]; (D) [94]; (E) [95]; (F) [96]; (G) [97]; (H) [98]; (I) [99]; (J) [100]; (K) [101]; (L) [102]; (M) [103]; (N) [104]; (O) [105]; (P) [106]; (Q) [107]; (R) [108]; (S) [109]; (T) [110]; (U) [111]; (V) [112]; (W) [113]; (X) [114]; (Y) [115]; (Z) [116]; (Aa) [117]; (Bb) [118]; (Cc) [119]; (Dd) [120]; (Ee) [121]; (Ff) [122]; (Gg) [70]; Hh) [123]; (Ii) [124]; (Jj) [125]; (Kk) [126]
